# Formulation and validation of a baseline prognostic score for osteosarcoma treated uniformly with a non-high dose methotrexate-based protocol from a low middle income healthcare setting: a single centre analysis of 594 patients

**DOI:** 10.3389/fonc.2023.1148480

**Published:** 2023-04-28

**Authors:** Shuvadeep Ganguly, Archana Sasi, Shah Alam Khan, Venkatesan Sampath Kumar, Love Kapoor, Mehar Chand Sharma, Asit Mridha, Adarsh Barwad, Sanjay Thulkar, Deepam Pushpam, Sameer Bakhshi

**Affiliations:** ^1^ Department of Medical Oncology, Dr. B.R.A. Institute Rotary Cancer Hospital, All India Institute of Medical Sciences, New Delhi, India; ^2^ Department of Orthopaedics, All India Institute of Medical Sciences, New Delhi, India; ^3^ Department of Pathology, All India Institute of Medical Sciences, New Delhi, India; ^4^ Department of Radiodiagnosis, Dr. B.R.A. Institute Rotary Cancer Hospital, All India Institute of Medical Sciences, New Delhi, India

**Keywords:** prognostic, score, osteosarcoma, low middle income countries, bone sarcoma

## Abstract

**Introduction:**

The outcomes of osteosarcoma in low middle income countries (LMICs) are different due to patients presenting in advanced stages, resource constraints and the use of non-high-dose-methotrexate (HDMTX)-based regimens. This study derived and validated a prognostic score for osteosarcoma that integrates biologic and social factors and is tailored for patients from an LMIC setting using a non-HDMTX-based protocol.

**Materials and methods:**

A retrospective study including osteosarcoma patients enrolled for treatment at a single tertiary care centre in India between 2003-19 was conducted. Baseline biologic and social characteristics were extracted from medical records and survival outcomes were noted. The cohort was randomised into a derivation and validation cohort. Multivariable Cox regression was used to identify baseline characteristics that were independently prognostic for survival outcomes in the derivation cohort. A score was derived from the prognostic factors identified in the derivation cohort and further validated in the validation cohort with estimation of its predictive ability.

**Results:**

594 patients with osteosarcoma were eligible for inclusion in the study. Around one-third of the cohort had metastatic disease with 59% of the patients residing in rural areas. The presence of metastases at baseline (HR 3.39; p<0.001; score=3), elevated serum alkaline phosphatase (SAP) >450 IU/L (HR 1.57; p=0.001; score=1) and baseline tumour size > 10 cm (HR 1.68; p<0.001; score=1) were identified to be independent factors predicting inferior event free survival (EFS) and were included in development of the prognostic score. Patients were categorized as low risk (score 0), intermediate risk (score 1-3) and high risk (4-5). Harrell’s c-indices for the score were 0.682, 0.608 and 0.657 respectively for EFS in the derivation, validation and whole cohort respectively. The timed AUC of ROC was 0.67 for predicting 18-month EFS in the derivation, validation and whole cohorts while that for 36-month EFS were 0.68, 0.66 and 0.68 respectively.

**Conclusions:**

The study describes the outcomes among osteosarcoma patients from an LMIC treated uniformly with a non-HDMTX-based protocol. Tumor size, baseline metastases and SAP were prognostic factors used to derive a score with good predictive value for survival outcomes. Social factors did not emerge as determinants of survival.

## Introduction

Osteosarcoma is the most common bone sarcoma worldwide (1, 2). The survival rates for bone sarcomas have improved over the last two decades on account of the incorporation of multi-modality treatment regimens. However, treatment outcomes continue to lag behind in low and low middle income countries (LMICs) due to a multitude of factors (2, 3). In LMICs, patients tend to present at advanced stages with high disease burden at presentation. Furthermore, healthcare accessibility, surgical expertise, access to good supportive care, treatment abandonment rates and compliance to treatment remain poorer in LMICs (4, 5). While high-dose-methotrexate(HDMTX)-based protocols have become the standard chemotherapy regimens in resource-rich settings, the delivery of HDMTX-based regimens entails logistic difficulties in the form of need for inpatient admission and strong supportive care, thus necessitating the use of alternate strategies in settings with resource limitations (6). Thus, treatment outcomes and their determinants are likely to be different in LMICs.

The identification of prognostic factors at baseline may facilitate tailoring of therapy based on disease risk. Prior studies have explored prognostic factors for survival in osteosarcoma. Baseline clinical factors such as extremes of age, large tumour sizes, axial tumour site as opposed to appendicular, and the presence of metastases have been found to be associated with worse survival outcomes (7–12). In addition, baseline lab parameters such as the neutrophil-lymphocyte ratio and alkaline phosphate have also been described to be of prognostic significance (13, 14). Among tissue immunohistochemistry markers assessed for prognostic value, tumour vascular endothelial growth factor (VEGF) response to neoadjuvant therapy has been noted to predict for more aggressive disease biology, while tumour HER2/neu expression was not found to be prognostic (15, 16).Imaging response surrogates using ^18^F-fluorodeoxyglucose positron emission tomography, computed tomography (^18^F-FDG PET-CT) and dynamic contrast enhanced magnetic resonance imaging (DCE-MRI) have been evaluated as markers for response to neoadjuvant chemotherapy (17, 18). Patients with poor histopathologic response to neoadjuvant therapy have been described to have inferior treatment outcomes (8, 19, 20). However, the intensification of therapy based on necrosis has not been conclusively shown to improve survival, especially among patients receiving HDMTX-based protocols (21). Therapy intensification based on baseline perceived disease risk has not been attempted previously on a background of chemotherapy protocols used in the current era (22, 23). The studies from which prognostic markers have been identified are largely registry based or have evaluated patients enrolled in large randomised controlled trials, which may not be reflective of the real world scenario. Furthermore, there is a striking lack of data from LMICs on therapeutic outcomes in osteosarcoma, wherein treatment protocols and the challenges involved in implementing them are unique.

In resource-challenged settings, social factors are also significant contributors to treatment outcomes. We have previously seen that the magnitude of gender disparity in seeking treatment for childhood cancer was dependent on the cost involved (24). Studies from the West have noted that social factors such as socioeconomic status and the possession of health insurance may be major determinants of survival in osteosarcoma (25, 26). Since the influence of social factors is likely to be more apparent in an LMIC setting, it is of great importance to identify their contribution to treatment outcomes along with tumor-related biological factors.

This study was conducted to derive and validate a prognostic score based on baseline disease characteristics along with analysis of impact of social characteristics on outcome in patients with osteosarcoma in an LMIC setting treated uniformly using a non-HDMTX based regimen. This may allow clinicians in LMICs to better risk stratify and tailor treatment based on the distinctive characteristics of patients with osteosarcoma hailing from more resource-challenged parts of the world.

## Methods

### Study design

This is a retrospective study from a single tertiary care cancer centre in India. Consecutive patients registered in the period between 2003 to 2019 in the medical oncology outpatient department were included. All patients included had a histopathologic diagnosis of osteosarcoma confirmed based on characteristic morphologic features seen on the biopsy specimen and discussion in the interdisciplinary conference. Patients who had received chemotherapy outside prior to or after presentation to our centre and those lost to follow up after receiving less than two cycles of (neo)adjuvant chemotherapy at our centre were excluded. Ethics approval was taken from the institute ethics committee (IEC-454/06.05.2022, RP-34/2022). In view of the retrospective nature of the study, the need for informed consent was waived off.

### Data collection

For all included patients, treatment files were reviewed to collect baseline data. Telephonic follow up was done to enhance data retrieval for patients with missing data and for those who were lost to follow up. Baseline clinical characteristics such as age, gender, symptom duration prior to presentation, presence of fever, clinical evidence of neurovascular bundle involvement, tumour size and disease stage were recorded. The baseline lab parameters compiled included hemogram and liver and renal function tests including serum alkaline phosphatase. The social characteristics comprised distance of the patient’s residence from the treating centre and the type of residence (rural versus urban). GoogleMaps was used to derive the distance of the treating centre from the address (27). The place of residence was categorised as rural or urban based on the address as per the National Census 2011 (28). Patients with metastatic disease were classified as “limited burden metastases” if they had two or fewer lung metastases and those with 3 or more lung metastases or any extrapulmonary metastases were classified as “extensive metastases”.

### Evaluation of the patient at baseline

All patients with confirmed diagnosis of osteosarcoma availing treatment at our institute were subjected to a standard set of baseline investigations prior to initiation of treatment. Imaging of the local site was done with MRI (magnetic resonance imaging). Baseline staging was done using either ^18^F-FDG PET-CT of the whole body or with a combination of non-contrast computed tomography (NCCT) of the thorax and a 99m-technetium methylene diphosphonate (Tc-99m MDP) bone scintigraphy.

### Treatment protocol

All patients were treated with a uniform non-HDMTX-based chemotherapy protocol. Three cycles of neoadjuvant therapy with cisplatin and doxorubicin were administered following which therapy response was evaluated with the help of local and distant site imaging. The RECIST 1.0 criteria were used for response assessment. Local therapy was planned after multidisciplinary discussion with the surgical team. The histopathologic response to neoadjuvant therapy was assessed based on necrosis in the postoperative specimen. Patients showing good responses (necrosis > 90%) were given three cycles of adjuvant chemotherapy with cisplatin and doxorubicin; on the other hand, patients with poor responses (necrosis < 90%) were given three alternating cycles each of cisplatin/doxorubicin and ifosfamide/etoposide as adjuvant chemotherapy (19, 29, 30). In patients with lung metastases at baseline, patients with partial or complete responses following neoadjuvant chemotherapy were considered for lung metastasectomy. Patients with disease progression at the metastatic site(s) were managed further with palliative intent.

### Outcomes of the study

The primary outcome in our study was event free survival (EFS) and the secondary outcome was overall survival (OS). The EFS was defined as the time between initiation of treatment and either disease progression or death from any cause. OS was defined as the time between treatment initiation and death from any cause. The data was censored on 30 November 2022.

### Statistical analysis

Statistical analysis was done with the help of STATA v.17 (StataCorp, College Station, TX, USA). Descriptive statistics was used to summarize baseline characteristics. Continuous variables were represented by median with range. The chi-square test and Mann-Whitney test were used to compare categorical and continuous variables respectively, and Kaplan Meier analysis was done along with log rank test to compare time to event outcomes. The follow-up estimation of the cohort was done using reverse Kaplan Meier method. The association of social factors [distance from treating centre (>100 km versus < 100 km) and type of residence (rural versus urban)] with baseline clinical characteristics was analysed by the chi-square test while the impact of social factors on survival outcomes was analysed by the log rank test. The impact of burden of metastases (limited versus extended burden metastases) on survival was also analysed by the Kaplan Meier and Cox regression methods.

### Generation of the derivation and validation cohorts and identification of prognostic factors in the derivation cohort

The whole cohort was divided in a 2:1 ratio into a derivation and validation cohort in a randomised fashion. The baseline factors assessed as potential prognostic factors included age (>18 vs ≤ 18 years), gender, symptom duration prior to presentation (>4 months vs ≤ 4 months), presence of fever, disease stage (localised versus metastatic), tumour size (>10 cm vs ≤ 10 cm), tumour site (axial vs appendicular), clinical presence of neurovascular bundle involvement, haemoglobin (<11 g/dL vs ≥ 11 g/dL), total leucocyte count (≤11000/µL vs >11000/µL), serum albumin (≥3.5 g/dL vs < 3.5 g/dL), serum alkaline phosphatase (>450 IU/L vs ≤ 450 IU/L). Univariable cox regression analyses were used to identify baseline factors prognostic for EFS in the derivation cohort. Factors with p-value less than 0.1 on univariable analyses were included for multivariable analysis in a forward stepwise fashion based on likelihood ratio. Factors with p<0.05 in the final multivariable model in the derivation cohort were used to formulate the risk score.

### Formulation of risk score

A weighted score was provided to each prognostic variable. The score was computed based on the approximate ratios of the beta coefficients of each factor in the multivariable model. The total score was calculated by summation of individual prognostic factor scores and was used to divide patients into three clinically discriminatory risk groups.

### Validation of the risk score

The risk score was validated by applying it separately to the derivation, validation, and whole cohorts separately. Kaplan Meier curves were constructed to represent EFS and OS in the three risk groups in each of the three cohorts. Harrell’s concordance index (c-index) was calculated for estimating the predictive ability of the risk category model for EFS and OS in the derivation, validation and whole cohorts. A receiver operating characteristic (ROC) curve was also constructed by comparing the predicted and actual 18-month and 36-month EFS and OS in each of the three cohorts and the timed area under the ROC curve (timed AUC) for the derivation, validation and whole cohort was estimated.

## Results

### Baseline patient characteristics and survival outcomes

During the study period from 2003 to 2019, a total of 640 patients with osteosarcoma registered at our centre with available data records were screened for inclusion in the study, out of which 594 patients were finally included for analysis (**Figure S1**). The baseline sociodemographic and clinical characteristics of the entire cohort are summarized in **Table 1**. The median age of presentation was 18 years (range: 2-71 years) with predominantly male patients (411/594; 69.2%) and a male to female ratio of 2.25:1. At presentation, the median tumor diameter (longest dimension) at the primary site was 10cm (range: 1-48 cm) with pathological fracture observed in 126 (21.4%) patients. Baseline metastatic disease was noted in more than one-third (204/594; 34.3%) of patients. At a median follow, up of 51.7 months (35.7-67.7 months), the median EFS of the whole cohort was 17.03 months while the estimated median OS was 80 months. The cohort was randomized 2:1 to yield 396 patients in the derivation cohort and 198 patients in the validation cohort. The baseline clinical and sociodemographic characteristics as well as the survival outcomes were similar between the two groups (**Table 1**).

**Table 1:** Baseline clinical and socio, demographic characteristics in derivation (n=396), validation (n=198) and whole cohort (n=594).

Clinical/Socio, demographic parameter (median with range)	Categories	Whole cohort (n=594)	Derivation cohort (n=396)	Validation cohort (n=198)	P-value*
* Clinical/demographic parameters					
1. Age (years)	Median (range)	18 (2 , 71)	18 (4, 66)	17 (2, 71)	0.499
	≤18 years	344 (57.9%)	225 (56.8%)	119 (60.1%)	
	> 18 years	250 (42.1%)	171 (43.2%)	79 (39.9%)	
2. Sex	Male	411 (69.2%)	270 (68.2%)	141 (71.2%)	0.451
	Female	183 (30.8%)	126 (31.8%)	57 (28.8%)	
3. Metastases	Non-metastatic	390 (65.7%)	265 (66.9%)	125 (63.1%)	0.359
	Metastatic	204 (34.3%)	131 (33.1%)	73 (36.9%)	
4. Tumor diameter of primary tumor (longest dimension) (cm) (n=482)	Median (range)	10 (1-48)	9.4 (1-48)	10.4 (2-29)	0.048
	≤10cm	270 (56.0%)	191 (59.3%)	79 (49.4%)	
	>10cm	212 (44.0%)	131 (40.7%)	81 (50.6%)	
5. Symptom duration (months) (n=502)	Median (range)	4 (1-36)	4 (1-36)	4 (1-36)	0.953
	≤4months	287 (57.2%)	190 (57.2%)	97 (57.1%)	
	>4months	215 (42.8%)	142 (42.8%)	73 (42.9%)	
6. Site of disease (n=525)	Axial	33 (6.3%)	20 (33.1%)	13 (7.5%)	0.431
	Appendicular	492 (93.7%)	331 (94.3%)	161 (92.5%)	
7. Fever at baseline	Yes	60 (10.1%)	34 (8.6%)	26 (13.1%)	0.083
	No	534 (89.9%)	362 (91.4%)	172 (86.9%)	
8. Fracture at presentation (n=590)	Yes	126 (21.4%)	85 (21.6%)	41 (20.8%)	0.820
	No	464 (78.6%)	362 (91.4%)	172 (86.9%)	
9. Neurovascular bundle involvement (n=582)	Yes	111 (19.1%)	67 (17.2%)	44 (22.9%)	0.098
	No	471 (80.9%)	323 (82.8%)	148 (77.1%)	
10. Hemoglobin (g/dL) (n=572)	Median (range)	11.7 (4 – 16.9)	11.8 (4-16.9)	11.5 (4.2-15.7)	0.182
	<11g/dL	208 (36.4%)	138 (36.1%)	70 (36.8%)	
	≥11g/dL	364 (63.6%)	244 (63.9%)	120 (63.2%)	
11. Total leucocyte count (/µL) (n=571)	Median (range)	8300 (990-42800)	8250 21-42800	8300 (990-24800)	0.930
	≤11000	481 (84.2%)	318 (83.7%)	163 (85.3%)	
	>11000	90 (15.8%)	62 (16.3%)	28 (14.7%)	
12. Serum Alkaline phosphatase (IU/L) (n=507)		452 (73 – 14960)	440 (73-14960)	496 (106-11550)	0.244
	≤450IU/L	271 (49.9%)	189 (51.8%)	82 (46.1%)	
	>450IU/L	272 (50.1%)	176 (48.2%)	96 (53.9%)	
13. Serum Albumin (g/dL) (n=531)		4.4 (2.0 – 6.2)	4.4 (2.1-6.2)	4.4 (2.0-5.6)	0.997
	<3.5g/dL	49 (9.2%)	29 (8.2%)	20 (11.4%)	
	≥3.5g/dL	482 (90.8%)	326 (91.8%)	156 (88.6%)	
* Sociodemographic parameters	Median(range)				
14. Distance from hospital (km) (n=537)		197 (20-2762)	177.5 (20-2005)	259(20-2762)	0.604
	≤100km	192 (35.8%)	138 (38.5%)	54 (30.2%)	
	>100km	345 (64.2%)	220 (61.5%)	125 (69.8%)	
15. Type of residence (n=537)	Urban	317 (59.0%)	208 (58.1%)	109 (60.9%)	0.535
	Rural	220 (41.0%)	150 (41.9%)	70 (39.1%)	
* Survival outcomes					
16. Mortality		217 (36.6%)	141 (35.6%)	76 (38.6%)	0.479
17. Median event free survival (months)		17.03 (14.8-19.2)	16.6 (13.3 – 20.1)	17.7 (14.4-20.9)	0.178
18. Median overall survival (months)		80 (estimate not reached)	Estimate not reached	55.7	0.820

*Continuous variables were reported as median with range. Median values between derivation cohort and validation cohort were compared during Mann-Whitney tests, while categorical variables between derivation cohort and validation cohort were compared using Chi-square test and similarly time to event outcomes were compared using log rank test.

### Identification of prognostic factors in the derivation cohort

**Figure 1:**
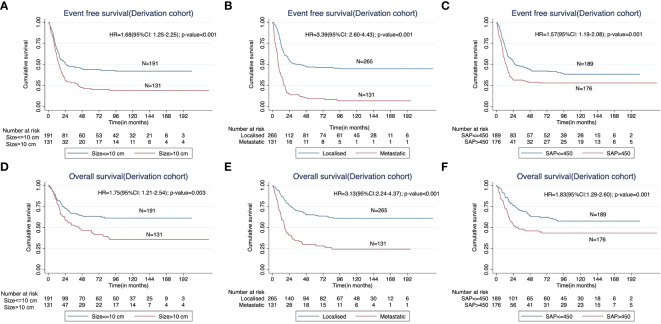
Kaplan Meier curves showing impact of **(A)** the baseline tumor size (< 10 cm versus > 10 cm), **(B)** presence of metastases at presentation, and **(C)** higher baseline serum alkaline phosphatase (≤450IU/L vs >450IU/L) on the event free survival (EFS) in the derivation cohort. The impact of the corresponding factors on overall survival (OS) is shown in **(D–F)**.

In the derivation cohort, on univariable analysis, the presence of baseline metastatic disease (HR=3.39; p<0.001); tumor diameter (longest dimension) >10cm (HR=1.68; p=0.005); neurovascular involvement at the primary site (HR=2.84; p<0.001); presence of a pathological fracture at baseline (HR=2.02;p<0.001); higher baseline serum alkaline phosphatase (>450 IU/L) (HR=1.57; p=0.001); and baseline anemia (hemoglobin < 11g/dL) (HR=1.38; p=0.021) were predictive of inferior EFS. However, on multivariable analysis, only the presence of baseline metastases (HR=3.55; p<0.001); tumor diameter >10cm (HR=1.38; p=0.045) and higher serum alkaline phosphatase (HR=1.50; 95%; p=0.010) were independently predictive of inferior EFS in the derivation cohort (**Table 2**; **Figures 1A–C**). The above three factors were also predictive of inferior OS in the derivation cohort. (**Figures 1D–F**).

**Table 2:** Univariable and multivariable analyses of prognostic factors for event free survival in the derivation cohort (n=396).

Prognostic factors	Categories (n)	Median event free survival (months)	Univariable analysis			Multivariable analysis*		
			HR	95% CI	P value	HR	95% CI	P-value
1. Age (years)	≤18 (225)	16.9	1	–	–	–	–	–
	>18 (171)	15.6	1.004	0.77, 1.31	0.974	–	–	–
2. Sex	Male (270)	16.1	1.24	0.93, 1.66	0.144	–	–	–
	Female (126)	19.6	1	–	–	–	–	–
3. Tumor diameter of primary site (Longest dimension)	≤10cm (191)	24.4	1	–	–	1	–	–
	>10cm (131)	14.9	1.68	1.25, 2.25	0.00049	1.38	1.01, 1.89	0.045
4. Site	Appendicular (331)	19.6	1	–	–	–	–	–
	Axial (20)	11.8	1.19	0.63, 2.27	0.578	–	–	–
5. Neurovascular involvement	Yes (67)	8.2	2.84	2.11, 3.81	<0.0001	–	–	–
	No (323)	22.5	1	–	–	–	–	–
6. Symptom duration	≤4 months (190)	18.1	1.26	0.94, 1.70	0.122	–	–	–
	>4 months (142)	24.4	1	–	–	–	–	–
7. Fever at baseline	Yes (34)	16.1	1.13	0.74, 1.74	0.575	–	–	–
	No (362)	16.9	1	–	–	–	–	–
8. Pathological fracture at baseline	Yes (85)	9.7	2.02	1.52, 2.69	<0.0001	–	–	–
	No (308)	21.4	1	–	–	–	–	–
9. Metastases at baseline	Yes (131)	8.3	3.39	2.60, 4.43	<0.0001	3.55	2.58, 4.88	<0.0001
	No (265)	37.5	1	–	–	1	–	–
10. Hemoglobin (g/dL)	<11 (138)	13.8	1.38	1.05, 1.81	0.021	–	–	–
	≥11 (244)	18.3	1	–	–	–	–	–
11. Total leucocyte count (/µL)	≤11000 (318)	17.8	1	–	–	–	–	–
	>11000 (62)	14.1	1.16	0.78, 1.60	0.552	–	–	–
12. Serum Albumin (g/dL)	≥3.5 (326)	16.6	1	–	–	–	–	–
	<3.5 (29)	16.9	1.26	0.77, 2.04	0.352	–	–	–
13. Serum Alkaline Phosphatase (IU/L)	≤450 (189)	26.3	1	–	–	1	–	–
	>450 (176)	13.6	1.57	1.19, 2.08	0.0014	1.50	1.10, 2.05	0.010

HR, Hazard Ratio; CI, Confidence interval; Hazard of reference category is represented as 1.

*Multivariable analysis was done including variables with p ≤ 0.1 in univariable analyses in a forward stepwise manner based on likelihood ratio and only significant variables (p<0.05) in the multivariable model was reported.

### Formulation of baseline prognostic risk categories

The three independent prognostic factors predicting inferior EFS in the derivation cohort were used to formulate a baseline prognostic risk score. Based on the ratio of beta-coefficient of the final multivariable Cox regression model, a weighted integer score was assigned to each prognostic factor: presence of metastases (score of 3); tumor diameter >10cm at primary site (score of 1) and baseline serum alkaline phosphatase >450IU/L (score of 1). Based on the scores, the patients were further categorized to clinically discriminatory risk categories (low risk: Score of 0; intermediate risk: score of 1,2 and 3; high risk: score of 4 and 5).

### Prognostic ability of the risk score category for event free survival

**Figure 2:**
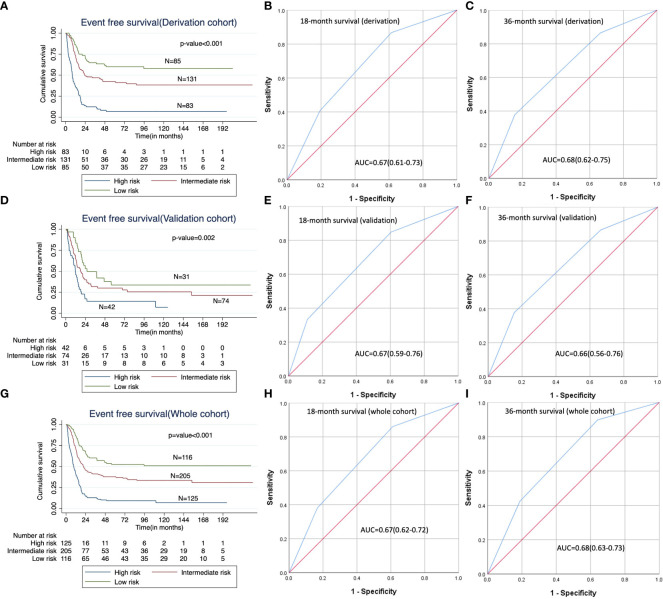
Predictive ability of the risk score category; **(A, D, G)**: Kaplan Meier curves showing impact of risk score category on EFS in the derivation, validation and whole cohorts respectively; **(B, E, H)**: Receiver operating characteristic (ROC) curves for the risk score categories for 18-month EFS in the derivation, validation and whole cohorts respectively; **(C, F, I)**: Receiver operating characteristic (ROC) curves for the risk score categories for 36-month EFS in the derivation, validation and whole cohorts respectively.

On application of the risk score to categorise patients in the validation cohort, the median EFS was significantly different among the three risk categories (median EFS of low risk, intermediate risk and high risk categories were 26.0 months versus 18.5 months versus 11.8 months respectively, log rank p-value=0.002). Similarly, the median EFS was significantly different among the three risk categories in both derivation (log rank p-value<0.001) and whole cohorts (log rank p-value<0.001). The estimated 18-month EFS in the low, intermediate and high risk categories in the validation cohort are 74 ± 8%, 50 ± 6% and 29 ± 8% respectively. The corresponding values for the 36-month EFS in the validation cohort are 49 ± 9%, 32 ± 6% and 14 ± 6% respectively in the three risk groups. The 18-month and 36-month EFS values as estimated in the derivation and whole cohorts are shown in **Table S1**. The Harrell’s c-indices of the risk score category for EFS in the derivation, validation and whole cohort were 0.682, 0.608 and 0.657 respectively. The timed AUC of ROC for predicting 18-month EFS in the derivation, validation and whole cohort were 0.67 (0.61-0.73), 0.67 (0.59-0.76) and 0.67 (0.62-0.72) respectively, while that of 36-month EFS in the derivation, validation and whole cohort were 0.68 (0.62-0.75), 0.66 (0.56-0.76) and 0.68 (0.63-0.73) respectively. (**Table S1** and **Figure 2**).

### Prognostic ability of the risk score category for overall survival

On application of the risk score category in the validation cohort, the median OS in the three categories was significantly different (median OS in the low risk, intermediate risk and high risk categories were 66 months versus 53.6 months versus 18.8 months, log rank p-value=0.027). (**Table S1** and **Figure S2**). Similarly, the median OS was significantly different among the three risk categories in both the derivation (log rank p-value<0.001) and the whole cohort (log rank p-value<0.001) as well. The estimated 18-month OS in the low, intermediate and high risk categories in the validation cohort are 90 ± 5%, 79 ± 5% and 55 ± 9% respectively. The corresponding values for the 36-month OS in the validation cohort are 70 ± 9%, 63 ± 6% and 35± 9% respectively in the three risk groups. The 18-month and 36-month OS values in the derivation and whole cohorts are shown in **Table S1**. The estimated 18-month and 36-month OS in the derivation, validation and whole cohort are shown in **Table S1**. The Harrell’s c-indices of the risk score category for OS in the derivation, validation and whole cohort were 0.681, 0.603 and 0.654 respectively. The timed AUC of ROC values for predicting 18-month OS in the derivation, validation and whole cohort were 0.68 (0.62-0.74), 0.68 (0.59-0.77) and 0.67 (0.63-0.73) respectively, while that for 36-month OS in the derivation, validation and whole cohort were 0.66 (0.60-0.73), 0.63 (0.54-0.73) and 0.66 (0.61-0.71) respectively. (**Table S1** and **Figure S2**).

### Impact of burden of metastases on survival

Among the 204 patients with metastatic disease at baseline, 143 (70.1%) had lung-only metastases, 42 (20.6%) had lung and bone metastases, 15 (7.4%) patients had isolated bone metastases and 4 (2%) had other sites of metastases. In the metastatic cohort, 56 patients (27.5%) had limited burden metastases while 148 patients (72.5%) had extensive metastases. It was seen that EFS in patients with limited burden metastases was significantly better than that of patients with extensive metastases (HR 0.62; p=0.007) but worse than that of patients with localised disease. (HR 2.2; p<0.001). However, OS of the cohort with limited metastatic burden was similar to patients with localised disease (HR 1.39; p=0.183) (**Figure S3**). Metastasectomy of lung metastases was done in 10 (5.15%) of 194 patients in the upfront setting.

In our patient cohort, 198 patients progressed after first line therapy. This included 23(11.6%) local-only recurrences, 118 (59.6%) isolated lung metastases, 40 (20.2%) patients with lung and local site recurrences, 8 patients (4.04%) with isolated bone metastases and 32 (16.2%) patients with metastases at other/multiple sites. Among the 161 patients having lung metastases at first relapse, metastasectomy was done for 48 patients (29.81%).

### Sociodemographic factors and their impact on baseline clinical factors and survival outcomes

In this study, the patients predominantly hailed from an urban residence (317/594; 59.0%) with median distance from the hospital of 197 km (20 to 2762 km), with similar distribution in the derivation and validation cohorts. The impact of residence and distance from the hospital on baseline clinical factors and survival outcomes is summarized in **Table 3**. The primary residence of the patient and the distance of the residence from the hospital were not predictive of either EFS or OS in the whole cohort. However, on multivariable analysis, patients with primary urban residence were more likely to have baseline tumor size greater than 10 cm (48.1% vs 37.5%, multivariable odds ratio 1.69; 95% CI: 1.13, 2.53, p=0.011) and less likely to have elevated total leucocyte count of more than 11000/µL (13.1% vs 20.0%; multivariable odds ratio 0.54; 95% CI: 0.31, 0.92; p=0.023). None of the remaining tumor characteristics or laboratory parameters significantly differed based on the type of primary residence or distance from the hospital (**Table 3**).

**Table 3:** Impact of sociodemographic parameters on clinical factors at presentation and survival outcomes of osteosarcoma in the whole cohort.

Parameter	Categories	Type of primary residence (n=537)			Distance of residence from hospital (n=537)		
		Urban residence (n=317)	Rural residence (n=220)	P value	≤100 km (n=192)	>100km (n=345)	P value
1. Age (years)	≤18	197 (62.1%)	133 (60.5%)	0.692	120 (62.5%)	210 (60.9%)	0.710
	>18	120 (37.9%)	87 (39.5%)		72 (37.5%)	135 (39.1%)	
2. Sex	Male	217 (68.5%)	157 (71.4%)	0.471	128 (66.7%)	246 (71.3%)	0.263
	Female	100 (31.5%)	63 (28.6%)		64 (33.3%)	99 (28.7%)	
3. Tumor size at primary site (longest dimension)	≤10cm	135 (51.9%)	110 (62.5%)	0.029	92 (58.2%)	153 (55.0%)	0.518
	>10cm	125 (48.1%)	66 (37.5%)		66 (41.8%)	125 (45.0%)	
4. Site of primary tumor	Axial	14 (5.0%)	14 (7.0%)	0.364	9 (5.5%)	19 (6.0%)	0.804
	Appendicular	266 (95.0%)	187 (93.0%)		156 (94.5%)	297 (94.0%)	
5. Symptom duration	≤4 months	150 (56.8%)	110 (59.8%)	0.532	104 (63.8%)	156 (54.7%)	0.061
	>4 months	114 (43.2%)	74 (40.2%)		59 (36.2%)	129 (45.3%)	
6. Neurovascular bundle involvement	Yes	59 (18.9%)	33 (15.3%)	0.290	37 (19.9%)	55 (16.1%)	0.277
	No	253 (81.1%)	182 (84.7%)		149 (80.1%)	286 (83.9%)	
7. Fever at baseline	Yes	33 (10.4%)	22 (10.0%)	0.878	20 (10.4%)	35 (10.1%)	0.921
	No	284 (89.6%)	198 (90.0%)		172 (89.6%)	310 (89.9%)	
8. Pathological fracture at baseline	Yes	64 (20.4%)	40 (18.3%)	0.544	42 (22.1%)	62 (18.1%)	0.261
	No	250 (79.6%)	179 (81.7%)		148 (77.9%)	281 (81.9%)	
9. Metastases at baseline	Yes	110 (34.7%)	75 (34.1%)	0.884	67 (34.9%)	118 (34.2%)	0.871
	No	207 (65.3%)	145 (65.9%)		125 (65.1%)	227 (65.8%)	
10. Hemoglobin (g/dL)	<11	111 (36.3%)	80 (38.1%)	0.674	65 (35.1%)	126 (38.1%)	0.508
	≥11	195 (63.7%)	130 (61.9%)		120 (64.9%)	205 (61.9%)	
11. Total leucocyte count (/µL)	≤11000	265 (86.9%)	168 (80.0%)	0.036	158 (85.9%)	275 (83.1%)	0.407
	>11000	40 (13.1%)	42 (20.0%)		26 (14.1%)	56 (16.9%)	
12. Serum Albumin (g/dL)	<3.5	28 (10.1%)	19 (9.5%)	0.840	18 (10.5%)	29 (9.5%)	0.721
	≥3.5	249 (89.9%)	180 (90.5%)		153 (89.5%)	276 (90.5%)	
13. Serum Alkaline phosphatase (IU/L)	≤450	133 (46.8%)	109 (53.2%)	0.166	87 (49.4%)	155 (49.5%)	0.985
	>450	151 (53.2%)	96 (46.8%)		89 (50.6%)	158 (50.5%)	
14. Median event free survival (months)		19.1 (15.5, 22.7)	16.9 (12.1, 21.7)	0.987	19.6 (15.5, 23.8)	17.0 (13.2, 20.7)	0.914
15. Median overall survival (months)		Estimate not reached	64.7 (Estimate not reached)	0.359	59.4 (Estimate not reached)	Estimate not reached	0.556

## Discussion

In this study, we analysed a retrospective cohort of osteosarcoma patients treated at our centre using a uniform non-HDMTX-based protocol. We formulated and validated a prognostic score based on baseline clinical factors and tailored to a unique population of patients treated in a resource constrained setting with a non-HDMTX-based protocol. Our survival outcomes were similar to those reported in smaller studies from LMICs but still lags behind those reported from Western countries (8, 20, 31, 32).

We identified metastases, tumour size and serum alkaline phosphatase to be important determinants of survival. The presence of metastases is a universally established prognostic factor in osteosarcoma (33). We observed that patients with limited burden metastatic disease had better EFS than those with extensive burden metastatic disease. It has been previously observed that osteosarcoma presenting only with lung metastases has better survival outcome than metastases at other sites (34). However, in our cohort, the proportion of patients ultimately undergoing metastasectomy remained low compared to eligible patients, which may be partially owing to resource limitations inherent to an LMIC setting. This exemplifies the need for better interdisciplinary coordination for implementing uniform protocols for metastasectomy for patients with limited number of lung metastases.

Large size and elevated alkaline phosphatase are surrogate markers for tumour burden. Large tumour size may hinder the penetration of drugs, thereby reducing chemosensitivity. Consequently, it has been identified to be prognostic for response to therapy and survival in prior studies (8, 35). Serum alkaline phosphatase is an indicator of osteoblastic activity and thus, may be indicative of disease aggressiveness (36). The normalisation of alkaline phosphatase following completion of neoadjuvant therapy has been identified to be a predictor of better survival; however, this was not assessed in the current study (37). Biomarkers of a systemic pro-inflammatory state such as total leukocyte count and hypoalbuminemia in Ewing sarcoma and hypoalbuminemia in both Ewing and soft tissue sarcomas have been seen to have prognostic value (38–40). However, these factors do not appear to be major predictors of treatment outcomes in osteosarcoma. The difference may be a consequence of differences in tumour microenvironmental profiles in the two tumours (41).

The prognostic factors identified in our cohort were largely similar to those described in HDMTX-based protocols. There are only few retrospective studies assessing prognostic factors while using non-HDMTX-based regimens in LMICs (31, 32, 42, 43). An analysis of another patient cohort from India using the non-HDMTX-based OGS-12 protocol has described serum alkaline phosphatase as prognostic for survival (43). Histologic response to chemotherapy has been described to be predictive in the studies available from LMICs (32, 42, 43). Metastases at presentation, tumour site and type of surgery were additionally identified to be prognostic in a Brazilian treatment cohort (44). The smaller size of the cohorts described, the shorter durations of follow up and the incorporation of treatment-related factors makes it difficult to generalise their results. Multicentre collaborative individual patient level data compilation may further our understanding of osteosarcoma in LMICs.

We designed a disease risk score based on the prognostic factors identified which had good discriminative value for distinguishing between groups with different survival. The tools currently available for risk stratification in osteosarcoma are derived predominantly from registry databases, which are inherently heterogenous in terms of institutional practices and regimens used (45–48). Although data derived from major randomised trials has enriched our understanding of prognostic factors in osteosarcoma, treatment in the setting of a trial may be subject to bias introduced by patient selection and differences in patient care as compared to real world data, thus making extrapolation difficult (8, 20, 49). Most scores have incorporated treatment-related factors into their algorithm (46, 47, 50, 51). Since treatment decisions may be altered based on baseline characteristics, such scores may be difficult to interpret. Our score was derived from a uniform single institution cohort using only basic clinical and lab parameters at presentation to allow for better risk stratification and prognostication at baseline. Furthermore, it is the only score available that is uniquely tailored to the LMIC setting accounting for treatment constraints and social backgrounds.

In current practice, non-HDMTX-based protocols incorporating risk stratified therapy, risk assessment is based on neoadjuvant chemotherapy response. Thus, treatment escalation for high risk disease has only been practised at completion of neoadjuvant chemotherapy (30, 52). The identification of high risk patients based on baseline characteristics may allow us to better demarcate candidates for upfront intensified therapy. The use of multiple non-cross-resistant drugs at therapy initiation may allow for better tumour responses in the context of high risk disease (53). The outcomes observed in patients with metastatic disease of high risk disease based on the score formulated are demonstrably poor. Thus, the score may be used to demarcate a subset of patients who may benefit from a palliative approach with early treatment de-intensification to avoid therapy-related and consequent reductions in quality of life (54, 55).

Social barriers to healthcare accessibility may lead to delays in treatment-seeking and may adversely affect compliance. We observed that patients with urban residence were more likely to present with larger sized tumors with lower total leukocyte counts; however, it did not have any impact on survival outcomes. A study from a Western country observed that residing at greater distances from the treatment centre and in areas of high unemployment was associated with higher mortality rates among osteosarcoma patients (56). Although social factors were integrated into our model, they did not have any significant impact on survival outcomes. This is further affirmed by our prior observation in bone sarcomas, where even in the context of resource challenged settings, tumour biology is a stronger determinant of the diagnostic interval than social factors (57). In addition, it has been seen that therapy-related factors such as delay in time to surgery following neoadjuvant chemotherapy and delay in the completion of planned therapy may compromise treatment outcomes (58, 59). Thus, optimising the delivery of healthcare services may allow for further improvements in survival.

The study represents the largest single institutional dataset of patients treated with a uniform non-HDMTX-based protocol. Furthermore, it is the largest dataset derived from a single institutional cohort in Asia. It provides a tool that allows the clinician to use baseline clinical and laboratory characteristics for risk stratification. It integrates social factors with clinical characteristics to better characterise the disease from the perspective of a resource-challenged setting. However, our study has a few limitations. Compliance to treatment and socioeconomic status were not assessed separately; thereby, their roles as potential prognostic factors could not be studied. However, the social background provided by the place of residence and distance from the treating centre may possibly serve as their surrogates. In the future, prospective studies may be formulated that evaluate the role of risk stratified therapy based on baseline characteristics to further improve outcomes.

## Conclusion

This study describes a large single institutional series of patients with osteosarcoma from an LMIC treated with a uniform non-HDMTX-based protocol. Clinical factors prognostic for survival at baseline were identified and used to derive and validate a risk score for prognostication. Tumour biologic characteristics were found to supersede social factors as determinants of survival.

## Data availability statement

The raw data supporting the conclusions of this article will be made available by the authors, without undue reservation.

## Ethics statement

The studies involving human participants were reviewed and approved by Institute Ethics Committee, All India Institute of Medical Sciences, New Delhi, India. Written informed consent from the participants’ legal guardian/next of kin was not required to participate in this study in accordance with the national legislation and the institutional requirements.

## Author contributions

SG analysed data, interpreted results, and wrote the manuscript. AS conceptualized the study, compiled the data, interpreted results, and wrote the manuscript. DP, SK, VK, LK, MS, AM, AB and ST conceptualized the study, provided intellectual inputs, administrative support and edited the manuscript. SB conceptualized the study, provided administrative support, intellectual inputs, interpreted results, wrote, and edited the manuscript. All authors have reviewed and approved the final version of the manuscript. All authors contributed to the article and approved the submitted version.
