# Supplementary material for: Formulation and validation of a baseline prognostic score for osteosarcoma treated uniformly with a non-high dose methotrexate-based protocol from a low middle income healthcare setting: a single centre analysis of 594 patients
Source: Front Oncol. 2023 Apr 28;13:1148480. doi: 10.3389/fonc.2023.1148480 (PMC10175811; doi:10.3389/fonc.2023.1148480)
Supplement: Supplementary file 1 [file DataSheet_1.docx]

**Formulation and validation of a baseline prognostic score for osteosarcoma treated uniformly with a non-high dose methotrexate-based protocol from a low middle income healthcare setting: A single centre analysis of 594 patients**

**Supplementary Appendix**

**Supplementary Table S1:** Event free survival and overall survival outcomes according to prognostic risk categories in derivation, validation, and whole cohort

**Supplementary Figure S1:** Workflow of the study

**Supplementary Figure S2:** Predictive ability of the prognostic score on overall survival

**Supplementary Figure S3:** Impact of burden of metastases on survival outcome

**Table S1: Event free survival and overall survival outcomes according to prognostic risk categories in derivation, validation and whole cohort**

| Risk categories | Whole cohort (n=446) | | | | Derivation cohort (n=299) | | | | Validation cohort (n=147) | | | |
| --- | --- | --- | --- | --- | --- | --- | --- | --- | --- | --- | --- | --- |
|  | **Number*** | **Median event free survival (months)** | **18-month event free survival** | **36-month event free survival** | **Number*** | **Median event free survival (months)** | **18-month event free survival** | **36-month event free survival** | **Number*** | **Median event free survival (months)** | **18-month event free survival** | **36-month event free survival** |
| Overall | 446 | 18.6 (15.4-21.8) | 48±2% | 36±2% | 299 | 19.6 (14.8-24.3) | 48±3% | 38±3% | 147 | 17.8 (13.8-21.8) | 49±4% | 31±4% |
| Low risk (score 0) | 116 | Estimate not reached | 75±4% | 60±5% | 85 | Estimate not reached | 75±5% | 65±6% | 31 | 26.0 (6.6-45.3) | 74±8% | 49±9% |
| Intermediate risk (score 1,2,3) | 205 | 21.5 (16.1-27.0) | 54±4% | 41±4% | 131 | 23.3 (3.0-43.5) | 57±5% | 47±5% | 74 | 18.5 (11.5-25.3) | 50±6% | 32±6% |
| High risk (score 4,5) | 125 | 9.6 (6.9-12.2) | 25±4% | 13±3% | 83 | 8.2 (6.7-9.6) | 23±5% | 12±4% | 42 | 11.8 (8.1-15.8) | 29±8% | 14±6% |
|  | **Number*** | **Median overall survival (months)** | **18-month survival** | **36-month survival** | **Number*** | **Median overall survival (months)** | **18-month survival** | **36-month survival** | **Number*** | **Median overall survival (months)** | **18-month survival** | **36-month survival** |
| Overall | 446 | 86.6 (Estimate not reached) | 73±2% | 60±2% | 299 | Estimate not reached | 72±3% | 60±3% | 147 | 52.6 (31.4-73.7) | 75±3% | 61±4% |
| Low risk (score 0) | 116 | Estimate not reached | 92±3% | 79±4% | 85 | Estimate not reached | 93±3% | 82±5% | 31 | 66.0 (Estimate not reached) | 90±5% | 70±9% |
| Intermediate risk (score 1,2,3) | 205 | Estimate not reached | 70±4% | 64±4% | 131 | Estimate not reached | 77±4% | 65±5% | 74 | 53.6 (29.8-77.5) | 79±5% | 63±6% |
| High risk (score 4,5) | 125 | 17.9 (12.2-23.7) | 52±5% | 37±5% | 83 | 17.7 (12.1-23.2) | 50±6% | 38±7% | 42 | 18.8 (10.1-27.4) | 55±9% | 35±9% |

*Total scores are available for only those patients where data regarding all three prognostic factors (Presence of metastases, Size of tumor at primary site and baseline serum alkaline phosphatase) are available

**

Figure S1: Workflow of the study**


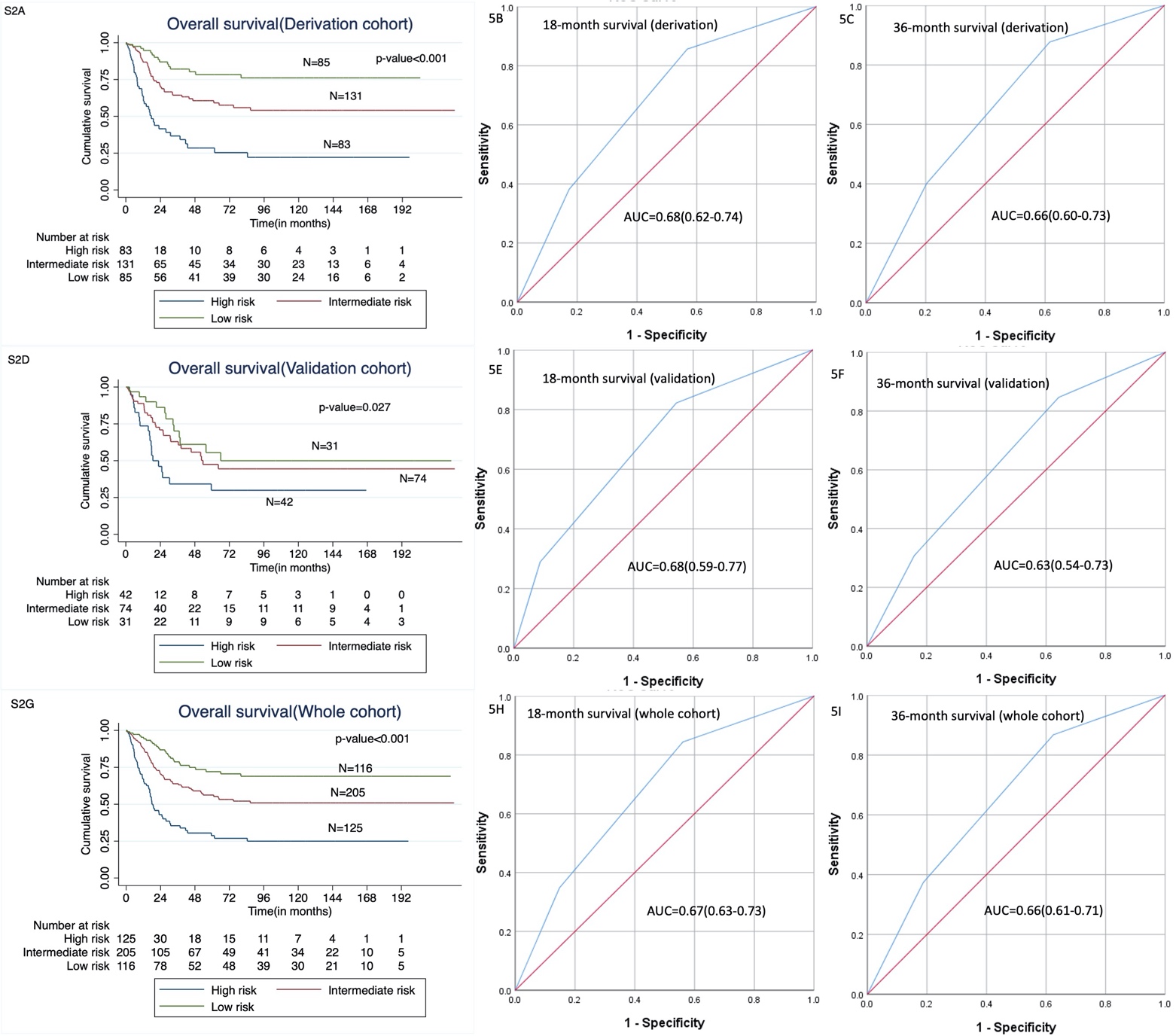


**Figure S2: Predictive ability of the prognostic score on overall survival**

**Figure S2:** Predictive ability of the risk score category on overall survival; **A, D, G**: Kaplan Meier curves showing impact of risk score category on OS in the derivation, validation and whole cohorts respectively; **B, E, H:** Receiver operator characteristic (ROC) curves for the risk score categories for 18-month OS in the derivation, validation and whole cohorts respectively; **C, F, I**: Receiver operator characteristic (ROC) curves for the risk score categories for 36-month OS in the derivation, validation and whole cohorts respectively

**Figure S3: Impact of burden of metastases on survival outcome
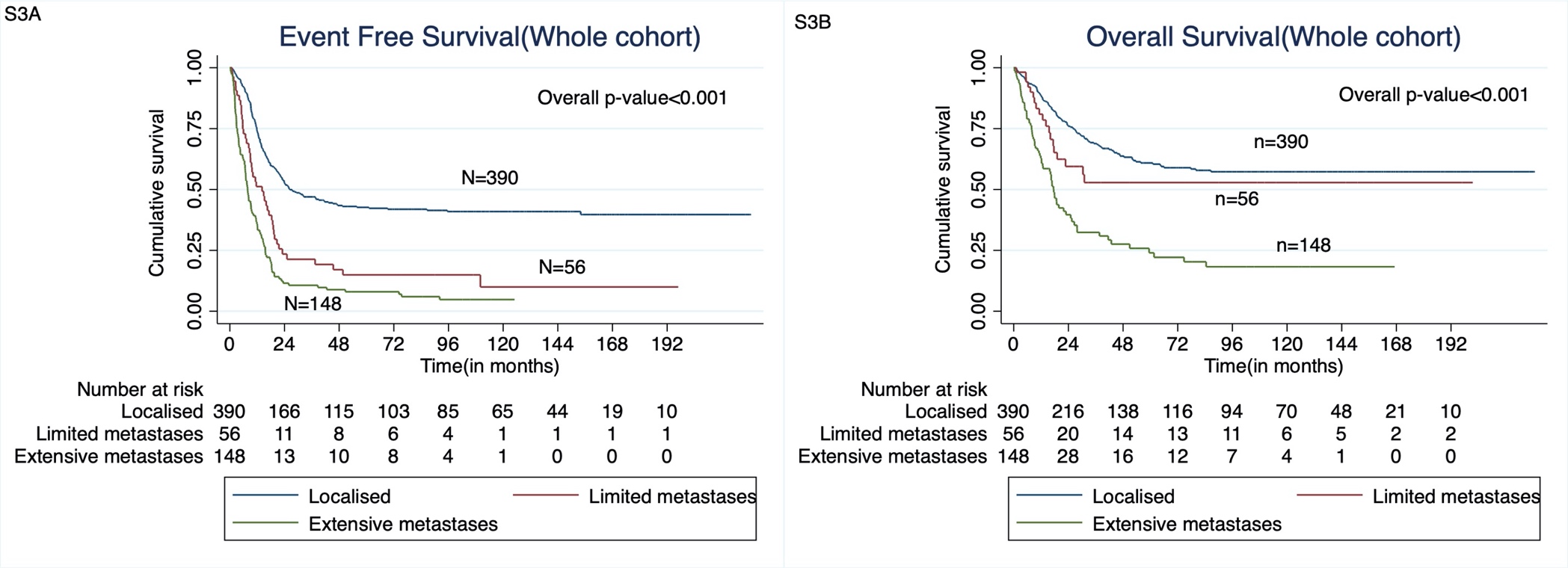
**

Figure S3: Kaplan Meier curves showing the impact of burden of metastases (localised disease versus limited burden metastases versus extensive burden metastases) on (A) event free (EFS) and (B) overall survival(OS)

For EFS, for limited burden versus localised disease: HR=2.21(95%CI: 1.59-3.06); p-value<0.001 and limited versus extensive burden disease: HR=0.62(95% CI: 0.44-0.87); p-value=0.007

For OS, for limited burden versus localised disease: HR=1.39(95%CI: 0.86-2.25); p-value=0.183 and limited versus extensive burden disease: HR=0.43(95% CI: 0.26-0.71); p-value=0.001
